# Reply to: Maternal capacity, twinning and fertility: the last birth matters

**DOI:** 10.1038/s41467-024-52549-2

**Published:** 2024-09-30

**Authors:** Alexandre Courtiol, Colin Vullioud, François Rousset, Erik Postma, Samuli Helle, Virpi Lummaa, Ritva Kylli, Jenni E. Pettay, Eivin Røskaft, Gine R. Skjærvø, Charlotte Störmer, Eckart Voland, Dominique Waldvogel, Ian J. Rickard

**Affiliations:** 1 Department of Evolutionary Genetics, Leibniz Institute for Zoo and Wildlife Research, Berlin, Germany; 2 Institut des Sciences de l’Évolution (ISEM), Université de Montpellier, CNRS, EPHE, IRD, Montpellier, France; 3 Center for Ecology and Conservation, University of Exeter, Penryn, UK; 4 INVEST Research Flagship Centre, University of Turku, Turku, Finland; 5 Department of Biology, University of Turku, Turku, Finland; 6 Department of History, University of Oulu, Oulu, Finland; 7 Department of Biology, Norwegian University of Science and Technology, Trondheim, Norway; 8 Institute for Philosophy, Justus Liebig University, Gießen, Germany; 9 Department of Evolutionary Biology and Environmental Studies, University of Zurich, Zurich, Switzerland; 10 Department of Anthropology, Durham University, Durham, UK

**Keywords:** Biological anthropology, Evolutionary ecology

**replying to** R. Meitern et al. *Nature Communications* 10.1038/s41467-024-52548-3 (2024)

To illuminate why twinning occurs in humans^1^, despite its threatening the health both of mothers^2^ and their children^3,4^, we analysed the relationship between twinning and fertility in a large, non-aggregated, multi-population, historical dataset of birth records from Northern and Central Europe^5^. Our analyses revealed a *negative* relationship between the probability that a mother produces more than one offspring per birth and her total number of births. This challenged the entrenched idea that mothers who are intrinsically more fertile—i.e., those who tend to conceive easily irrespective of age and other factors—show a physiological predisposition to produce twins (referred to as the “heterogeneity hypothesis” by us, and as the “maternal capacity hypothesis” by Meitern et al.^6^). In response to our work, Meitern et al.^6^ used a different, and exceptionally large, demographic dataset recently digitised by the Estonian Institute for Population Studies^7,8^ and successfully replicated some of our findings. However, after they discarded the last birth from each mother, they obtained a *positive* relationship between the per-birth twinning probability and total births. Meitern et al.^6^ interpreted this result as a novel support for the heterogeneity/maternal capacity hypothesis. Here we argue that differences between our studies^5,6^ are instead the result of the demographic transition—the shift from high to low fertility exhibited by all European populations over the 19^th^/20^th^ centuries—and that once this is accounted for, the two studies show strikingly similar results with neither of them supporting the heterogeneity/maternal capacity hypothesis.

To begin with the similarities, both studies find that—when all births by a woman are considered—the per-birth twinning probability is negatively related to the total number of births. Although Meitern et al.’s estimate of this relationship is less negative (Fig. 1a and Supplementary Table 2 in Meitern et al.^6^), it supports our conclusion^5,9^ that the strong *positive* relationship between the lifetime twinning status and total births documented in many different studies^5^ is likely to be due to an analysis performed at the wrong biological level (mothers rather than births) and not a finding in support of the heterogeneity/maternal capacity hypothesis. Meitern et al. furthermore confirmed our key finding that future reproduction is reduced after a twin delivery, for example, as a result of physiological impairment or family planning.

**Fig. 1:**
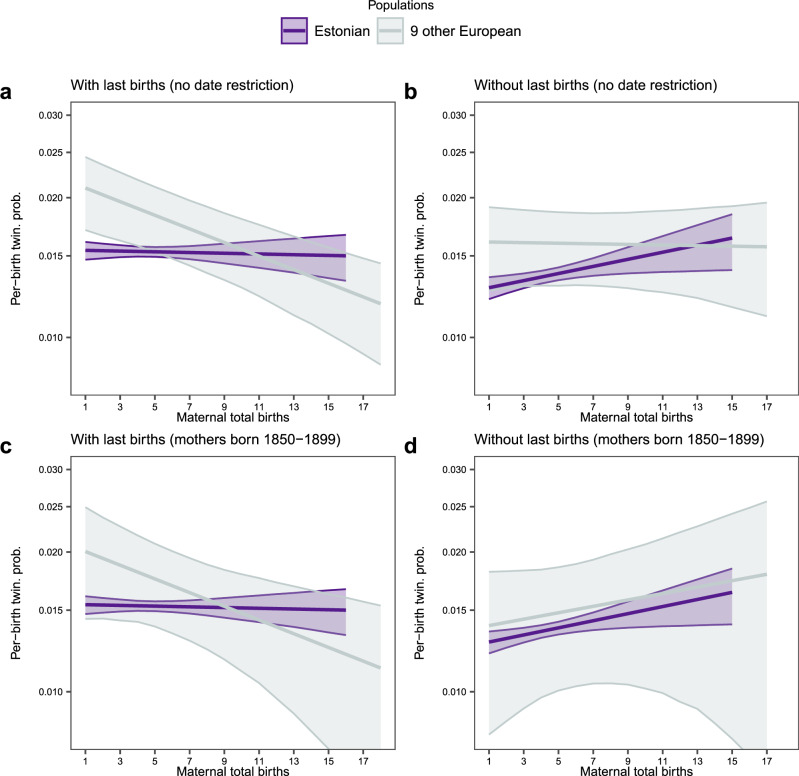
Relationship between per-birth twinning probability and maternal total births in nine European populations (grey) and a single Estonian population (purple) for different subsets of the data. In (**a**), no restrictions beyond the removal of 14 mothers with uncertain years of birth and general cleanup, as described in our paper^5^ were applied to the datasets, whereas in (**b**), the last births were discarded. To allow for a direct comparison of both samples, in (**c**) and (**d**), mothers born before 1850 were discarded from the nine European populations because no mothers were born before 1850 in the Estonian dataset. Whereas (**c**) includes all births, (**d**) is based on data excluding all last births. The number of birth events for the Estonian population is: 417,418 (**a**), 291,843 (**b**), 417,418 (**c**) and 291,843 (**d**), and the number of mothers for the Estonian population is 115,963 (**a**), 92,696 (**b**), 24,735 (**c**) and 19,325 (**d**). The number of birth events for the nine other populations is: 125,575 (**a**), 98,183 (**b**), 125,575 (**c**) and 98,183 (**d**), and the number of mothers for the nine other populations is: 23,267 (**a**), 20,309 (**b**), 5,410 (**c**) and 4,549 (**d**). Each plot shows marginal predictions (line) ± CI_95%_ (a grey area) from the fits of generalised linear mixed-effects models, including maternal total births as the fixed effect and, for the nine European populations, variation between populations as a random effect. The model structure is described in Eq. 3 in our paper^5^ with the modification that the random effect was dropped when fitting the Estonian data since those data only represent a single population. Data, computer code and details of the analysis can be found at https://github.com/courtiol/twinR.

As for discrepancies, Meitern et al.^6^ found that—if they removed the last births from their dataset—the relationship between per-birth twinning probability and total births became positive, whereas it remained negative in our multi-population study (Fig. 1b). However, the two datasets differ in an important way: whereas all mothers in the Estonian dataset are born after 1850, only 23.3% of the mothers in our dataset were. If we subsample our dataset to retain only mothers born during the same time period as those from the Estonian dataset, the discrepancy vanishes (Likelihood Ratio Test of the interaction between total births & dataset: χ^2^ < 0.01, df = 2, *p* ~ 1; Fig. 1d).

Two conclusions emerge from this finding. First, it shows that the relationship between per-birth twinning probability and total births is affected by the transition to modernity^10^. This result is in line with all mechanisms we hypothesised to shape the relationship between per-birth twinning probability and total births^5^ (i.e., parity progression, inter-birth intervals, the reproductive schedule of a mother, and maternal heterogeneity) having been impacted by the demographic transition^11–13^. Second, our multi-population study corroborates Meitern et al.‘s finding that the relationship between per-birth twinning probability and total births can be positive *after the removal of the last births*.

Understanding *why* the relationship between per-birth twinning probability and total births is what it requires disentangling and quantifying the effect of the mechanisms that may shape this relationship across a woman’s complete reproductive life. This is far from trivial because of how each of these factors may influence the multiple reproductive events of each mother in a non-linear fashion. To accommodate these complexities, we developed^5^ a goodness-of-fit analysis which combines statistical mixed-effects models fitted to real data with individual-based simulations. Meitern et al.^6^ applied this same framework to their data (after the removal of last births) to gain further insight into the relative role of heterogeneity/maternal capacity and other processes in explaining the relationship between per-birth twinning probability and total births.

First, they found that although mothers with high intrinsic fertility and twinning propensity exist, they represent one end of a continuum describing variation in maternal capacity between mothers (statistically represented by random effects; mechanism called “H” for heterogeneity in our paper^5^ and Supplementary Fig. 3 in Meitern et al.^6^); on the other end of this continuum are women for whom a higher twinning propensity is associated with a *lower* intrinsic fertility. In Rickard et al.^5^, we showed that along this continuum, there is a *negative* correlation between twinning propensity and other intrinsic fertility components (Supplementary Fig. 2 in the original study). Mothers with high intrinsic fertility and twinning propensity are thus rare, and mothers on the opposite end of the continuum prevail in our multi-population dataset. This explains why, similarly to what we documented, allowing for variation in per-birth twinning probability between individuals (at a given age and parity) in the analysis of the Estonian data (Supplementary Fig. 3 in Meitern et al.^6^) does not improve the goodness of fit of the simulation. Their results are thereby in line with our findings and together argue against the heterogeneity/maternal capacity hypothesis.

Second, the goodness-of-fit analysis of the Estonian data revealed that the positive relationship between per-birth twinning probability and total births is an emergent property of how age and parity impact these traits (called “S” for reproductive schedule). In fact, if that were the sole mechanism, the relationship would be slightly more positive than what was observed, and best fits are obtained when they take into account that fertility decreases after the birth of twins (called “P” for parity progression). In our study, which included all births, these two mechanisms were also the most important ones^5^.

In sum, while the positive relationship between per-birth twinning probability and total births that arises—after we remove the last births and limit ourselves to women born after 1850—may, at first sight, provide novel evidence for the heterogeneity/maternal capacity hypothesis, rigorous analysis of the available data does not support this intuition. Instead, the positive relationship is best reproduced by simulations involving mechanisms that do not assume differences in per-birth twinning probability between mothers other than those created by differences in mothers’ age and parity. Hence, neither study supports the existence of the “silver spoon” effect conjectured by Meitern et al.^6^, or any other mechanism that permanently affects maternal capacity (i.e., condition or quality) from the first birth (or earlier) onward.

Furthermore, and contrary to Meitern et al.^6^, discarding the last birth of each mother from the data is not “*an alternative approach for testing the prediction of the maternal capacity hypothesis*”. While we agree that doing so may reduce the effect of family planning on the total number of births, it does not help to test the focal hypothesis. Instead, it introduces peculiar biases. For example, it artificially reduces the twinning rate (the per birth twinning probability is highest for the last–that is, the removed–birth), and skews the sample toward non-twinners. It also removes all mothers that only reproduce once, and reduces parity (by one) as well as age at last reproduction. In short, it is not clear what a sample deviating so importantly from any real population represents. We thus caution against removing specific births before investigating the relationship between twinning and fertility. Instead, we encourage anyone interested in exploring complex scenarios other than the ones we considered to build on our statistical framework. Performing some form of inference by simulations is a necessary evil for the study of complex systems.

## Reporting summary

Further information on research design is available in the Nature Portfolio Reporting Summary linked to this article.

## Supplementary information

Reporting Summary
